# Psychometric evaluation of the Chinese version of the Toronto Hospital Alertness Test

**DOI:** 10.1186/s41687-020-00197-7

**Published:** 2020-05-05

**Authors:** Sha Li, Daniel Yee Tak Fong, Janet Yuen Ha Wong, Kate Wilkinson, Colin Shapiro, Edmond Pui Hang Choi, Bradley McPherson, Cindy Lo Kuen Lam, Mary Sau Man Ip

**Affiliations:** 1 School of Nursing, The University of Hong Kong, 21 Sassoon Road, Hong Kong, China; 2 Department of Psychiatry, University of Toronto, 399 Bathurst Street, Toronto, Canada; 3 Human Communication, Development, and Information Sciences, The University of Hong Kong, Pokfulam Road, Hong Kong, China; 4 Department of Family Medicine and Primary Care, The University of Hong Kong, 21 Sassoon Road, Hong Kong, China; 5 Department of Medicine, The University of Hong Kong, 21 Sassoon Road, Hong Kong, China

**Keywords:** Alertness, Confirmatory factor analysis, Reliability, Validity

## Abstract

**Background:**

Alertness is an important part of attention which is different from the opposite of sleepiness. This study aimed to translate and assess the measurement properties of the Toronto Hospital Alertness Test (THAT) in Hong Kong Chinese population.

**Methods:**

The standard forward-backward translation procedure and cognitive debriefing were conducted to obtain the Chinese THAT. One hundred Chinese adults completed the Chinese THAT, the Center for Epidemiological Studies Depression Scale (CES-D), the Pittsburgh Sleep Quality Index (PSQI), and the Athens Insomnia Scale (AIS) by telephone interviews.

**Results:**

The factorial validity was assessed by confirmatory factor analysis, and the internal reliability was examined by coefficient omega. The two negatively worded items of the THAT had low factor loadings and were removed. One more item was removed based on the modification indices of the eight-item model. The remaining seven-item THAT showed satisfactory unidimensionality with root mean square error of approximation (RMSEA) = 0.06, standardized root mean square residual (SRMR) = 0.08, and comparative fit index (CFI) = 1.00. The coefficient omega of the seven-item Chinese THAT was 0.80 (95% CI: 0.74–0.86). Convergent validity was demonstrated with THAT moderately associated with CES-D (*r* = − 0.45, *P* < 0.01), PSQI (*r* = − 0.40, *P* < 0.01), and AIS (*r* = − 0.45, *P* < 0.01).

**Conclusions:**

The Chinese version of THAT demonstrated acceptable reliability and validity in a Chinese population.

## Background

Attention, which in charge of information processing, is an important issue in education, psychology, and neuroscience studies [1]. Recently, researchers suggested investigating more specific components of attention, such as alertness, instead of the general process [1]. Alertness comprises of phasic alertness and tonic alertness [2]. Phasic alertness refers to the orienting response, and it changes relatively rapidly [2, 3]. Tonic alertness is equivalent to vigilance, as well as to sustained attention, and it changes relatively slowly [2, 3]. Alertness is commonly considered in medical sciences and is concerned with cognitive processing and is preferably assessed by physiological brain measurements [4], while it is described as the state of individual behavior with a focus on the interaction with the external or internal environment, such as sleep deprivation in behavioral sciences [5]. Many functionalists have resorted to define mental concepts functionally instead of philosophically or scientifically, which can make the concepts possible to be measured with external criteria [6]. Therefore, we refer to alertness as the responsivity to internal and external stimuli from the behavior approach in this article [6].

Low alertness levels may have an adverse impact on our daily life, but it has received limited attention in research. Impaired alertness is common not only in patients but also in the general population [7–9]. For example, truck drivers and health care workers are also at risk of impaired alertness [10–12]. It was estimated that 1.1 million crashes per year are associated with impaired alertness in the USA [12]. In addition to sleep deprivation, psychiatric disorders and prescribed or over-the-counter medications may also lead to impaired alertness [13, 14]. Impaired alertness may be associated with fatigue, low energy, drowsiness, reduced attention, and decreased concentration [9]. These symptoms and feelings can induce automobile accidents [15], psychological symptoms [16], physical symptoms [17], and decreased quality of life [9], as well as the higher likelihood of disability and increased risk of mortality [18]. In adolescents, daytime impaired alertness may induce depressed mood and impact academic performance, while depressed mood can reduce academic performance further [19].

A standardized measurement tool specific for alertness is needed to guide treatment and facilitate the development of psychopathology [6, 16]. Two instruments have been developed for assessing alertness, namely the Toronto Hospital Alertness Test (THAT) and the ZOGIM Alertness (ZOGIM-A) Scale [6]. THAT aims to assess self-perceived alertness, including the ability to concentrate, to think of new ideas, and to focus on the task at hand over the past week, whereas ZOGIM-A aims to evaluate self-perceived impact or benefits of alertness and the extent of experiencing high alertness [6]. Most researchers are concerned with alertness level and the negative effects of impaired alertness, making the THAT more frequently used than the ZOGIM-A. The THAT in original English language demonstrated satisfactory reliability and validity. The Cronbach’s coefficient alpha and test-retest reliability of THAT were 0.96 and 0.79, respectively. Acceptable convergent validity was also shown, with a significant correlation with ZOGIM-A (*r* = 0.37, *P* < 0.01) [6]. Although THAT is relatively new, it has already been used to evaluate the effect of interventions [20, 21] and also could distinguish self-reported anxious from non-anxious patients [16].

China is one of the most populous countries in the world. However, alertness has been less studied in Chinese populations, which may be partly attributed to the lack of a standardized measurement tool to assess alertness. Therefore, this study aimed to examine the psychometric performance of a Chinese language version of THAT in a Chinese population in Hong Kong.

## Methods

### Linguistic validation of the Chinese THAT

**Fig. 1:**
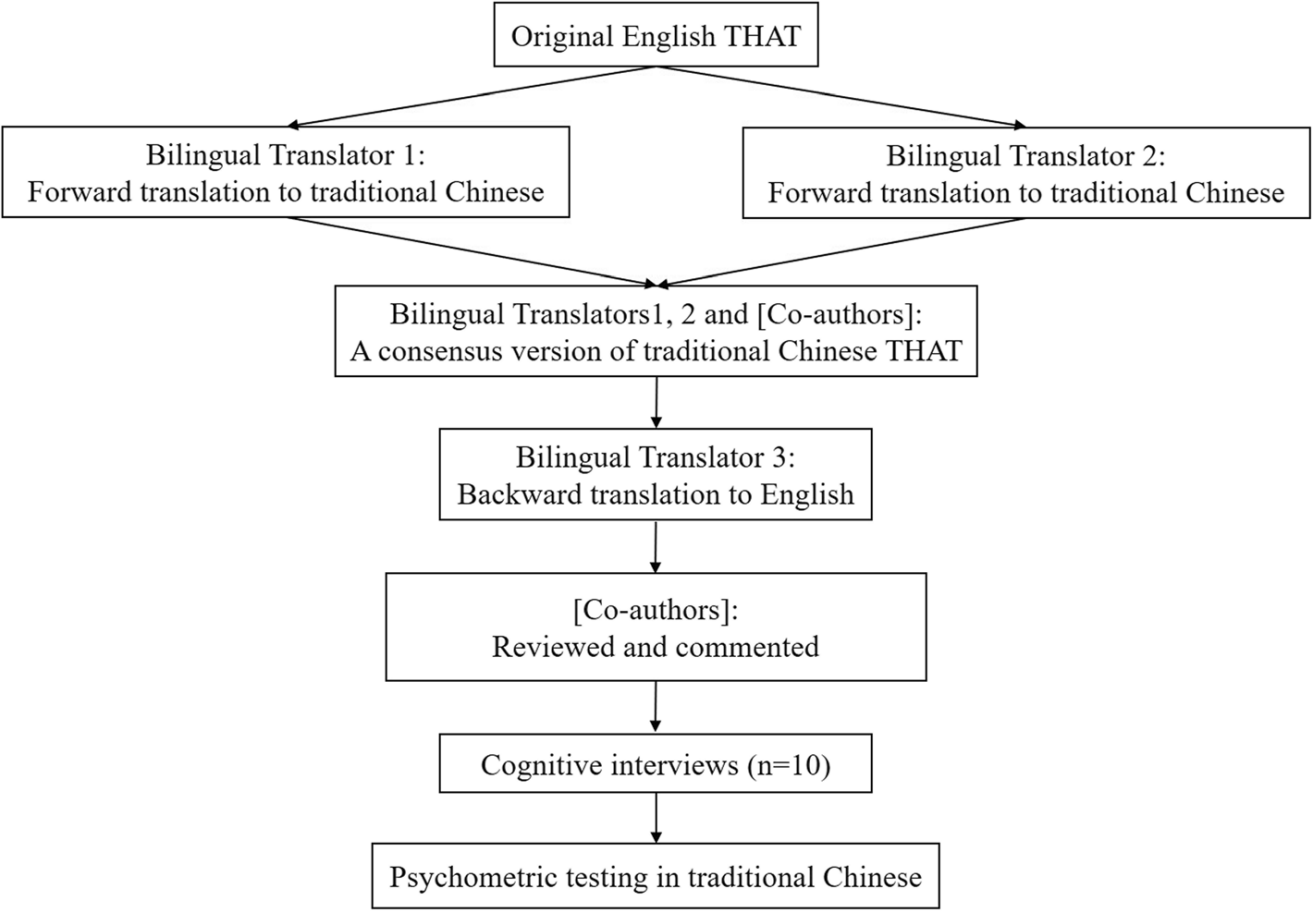
Linguistic validation procedure

Figure 1 shows the linguistic validation procedure. We developed the Chinese version of THAT from the original English version using standard forward-backward procedures [22]. The original developer of the THAT, a local academic sleep specialist in charge of a sleep clinic, an academic statistician with prior experience in linguistic and psychometric evaluation of patient-reported outcomes, and local registered nurses who were also fluent in English were invited to comprise the expert committee. Then, two bilingual registered nurses who were native Chinese independently translated the THAT into traditional written Chinese. The academic statistician hosted a meeting with the two forward translators to agree on a consensus version. Any discrepancies on the two Chinese versions were discussed and resolved by the statistician with consensus of the two translators, and the preliminary consensus version was then reviewed by an academic sleep specialist in charge of a sleep clinic. Further modifications on the wordings were made. The final consensus version was then back-translated into English by another registered nurse who was not informed of the original English version. Any discrepancies between the backward and the original English versions were assessed by the expert committee, and revision of the Chinese version was made where needed.

### Participants

One hundred subjects who were 18-year-old or above and were able to communicate in Chinese were targeted by telephone interview. Shift workers and those who were taking drugs for hypertension or sleep problems or who had mental illness were excluded. The THAT comprises 10 items. Using the standard estimate of 10 subjects per test item, 100 subjects would be adequate for assessing the construct validity of the Chinese version of THAT [23].

### Procedures

Participants were recruited in a telephone survey by random digit dialing of household residential fixed numbers. Each randomly selected phone number was added or minus one or two to capture unlisted numbers. When there was more than one eligible subject in a household, the occupant with the next birthday was explained the study details and invited to provide oral consent. If the subject consented to participate in the study, the participant would be interviewed in Cantonese using a questionnaire written in traditional Chinese characters. Ethical approval for the project was obtained from the Institutional Review Board of the University of Hong Kong/Hospital Authority Hong Kong West Cluster (Ref no.: UW16–326).

### Measures

#### Toronto hospital alertness test (THAT)

The 10-item THAT assessed self-perceived alertness over the past week [6]. Each item was graded on a 0–5 Likert scale. After reverse coding the final two test items, a global score was obtained as the response total. A higher global score corresponds to a higher level of alertness.

#### Center for Epidemiological Studies Depression Scale (CES-D)

The self-rated CES-D scale comprises 20 items that evaluate the depressive symptoms [24]. It has been shown to be a valid tool to screen subjects with depression and to evaluate the severity of depressive disorders in a Hong Kong Chinese population [25]. The items were responded to using a 0–3 Likert scale, according to the frequency of the symptoms. A higher score indicates more severe depression.

#### Pittsburgh sleep quality index (PSQI)

The 19-item PSQI questionnaire assesses sleep quality during the past month with diverse aspects relating to factors, including sleep latency and duration, subjective feeling of sleep quality and sleep efficiency, along with sleep-related problems [26]. The 19 items, each rated on a 0–3 scale, were grouped under seven components, namely: subjective sleep quality, sleep latency, sleep duration, habitual sleep efficiency, sleep disturbance, use of sleeping medications, and daytime dysfunction [26]. The component scores were the total scores of the corresponding items, which spans from 0 to 21 with a higher score indicating a worse sleep quality [27].

#### Athens insomnia scale (AIS)

The AIS is a self-report questionnaire for estimating sleep difficulty in the past month [28]. It contains eight items on sleep induction, awakenings during the night, final awakening earlier than desired, total sleep duration, the overall quality of sleep, as well as a sense of well-being and sleepiness during the day [29]. Participants graded their sleep quality from 0 to 3 according to the severity of their sleeping problems. The total score ranges from 0 to 24, with a higher score corresponding to a worse sleep quality [29]. The internal consistency of AIS in this study was 0.84.

### Statistical analysis

The Chinese version of THAT was scored as the English version. The last two items of the original 10-item scale were reversed scored, and the floor and ceiling effects were then checked before analysis. If an overall value of greater than 15% for ceiling or floor effect exists, the validity, reliability, and responsiveness of a scale would be affected [30]. The factorial validity was examined through confirmatory factor analysis (CFA) by testing root mean square error of approximation (RMSEA) values, standardized root mean square residual (SRMR), and comparative fit index (CFI). The cut-off values were selected as 0.06 or below, 0.08 or below, and 0.95 or higher for RMSEA, SRMR, and CFI, respectively [31]. In the CFA models, the responses were taken as ordinal variables, and the diagonal weighted least squares (DWLS) estimator was used. DWLS is considered superior to robust maximum likelihood (MLR) when analyzing ordinal variables in latent variable modeling [32]. Furthermore, items with factor loadings smaller than 0.4 were suggested to be removed [33]. If the number of item changes, floor and ceiling effects are evaluated again before proceeding with analyses. Reliability of the Chinese version of THAT was assessed by omega and corrected item-scale correlations. The omega is considered the best alternative to Cronbach’s α as the assumptions of Cronbach’s α, such as essentially tau-equivalence model, are usually violated [34]. The values of omega and corrected item-scale correlations were considered acceptable when greater than 0.7 [35] and 0.3 [23], respectively. When testing for convergent validity, for each subject with at most five (50%) non-responded items, the missing values were replaced with the average score of the remaining items. The Spearman rank correlation coefficients of the Chinese version of THAT with the CES-D, PSQI, and AIS were calculated to determine convergent validity. SPSS (version 23) and Rstudio-1.1.383 with the package “lavaan” [36] and “userfriendlyscience” [37] were adopted to perform data analysis. The significance level was set at 0.05.

## Results

### Demographic characteristics and Chinese version of THAT scores

We interviewed 100 subjects. Their average age was 61 years old (Standard deviation: 17, range: 18–88), and 43 (43%) were male. Twenty-five participants (25%) had primary education or below, 44 (44%) participants had secondary education, and 30 (30%) participants had a bachelor’s degree or above. There were 54 (54%) retired participants, 17 (17%) employees, 13 (13%) homemakers, 6 (6%) students, 4 (4%) employers, 3 (3%) self-employed participants, and 2 (2%) job-seeking participants.

Table 1 shows the item characteristics. In total, 95 (95%) participants completed all the items of THAT. Items 2, 7, and 8 had only one (1%) missing value, while items 3 and 10 had 2 (2%), and item 5 had 3 (3%) missing values. Despite high floor or ceiling percentages in some items, the overall 10-item scale score had only 1% ceiling and no floor effects.

**Table 1:** Summary of the Chinese version of THAT scores

Items	Missing values (%)	Mean (SD)	Min	Max	Median	Floor (%)	Ceiling (%)
1. Able to concentrate	0	4.0 (1.5)	0	5	5	4	57
2. Alert	1	3.7 (1.5)	0	5	4	7	45
3. Fresh	2	2.3 (2.0)	0	5	2	30	21
4. Energetic	0	2.9 (1.8)	0	5	3	17	27
5. Able to think of new ideas	3	1.6 (1.9)	0	5	1	44	14
6. Vision was clear noting all details (e.g., driving)	0	3.9 (1.6)	0	5	5	8	57
7. Able to focus on the task at hand	1	4.2 (1.2)	0	5	5	2	58
8. Mental facilities were operating at peak level	1	3.8 (1.4)	0	5	4	3	46
9. Extra effort was needed to maintain alertness	0	3.4 (2.0)	0	5	5	19	52
10. In a boring situation, I would find my mind wandering	2	3.8 (1.5)	0	5	4	7	44
10-item THAT	5	33.4 (9.7)	3	50	36	0	1
8-item THAT (items 9 and 10 deleted)	5	26.3 (8.6)	3	40	28	0	4
7-item THAT (items3, 9 and 10 deleted)	4	24.1 (7.4)	3	35	26	0	5

*SD* Standard deviation

### Factorial validity

The 10-item one-factor model of THAT did not show satisfactory fit. The factors loadings of Item 9 “Extra effort was needed to maintain alertness” and Item 10 “In a boring situation, I would find my mind wandering” were 0.29 and 0.39, respectively, both smaller than 0.4. These two items were then removed, and the resulting eight-item one-factor CFA model was assessed.

**Fig. 2:**
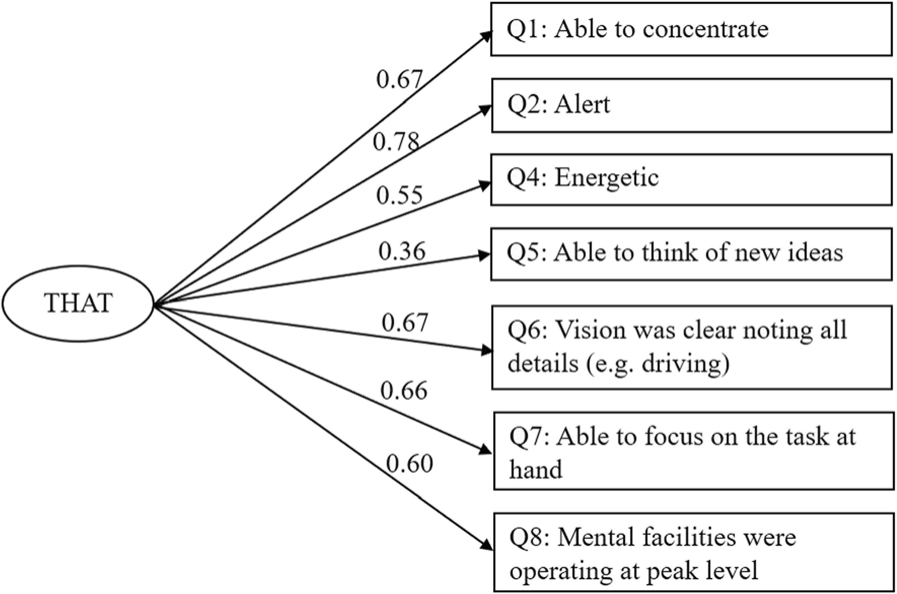
Standardized coefficients of the seven-item Chinese version of Toronto Hospital Alertness Test (THAT)

The eight-item scale score had 4% ceiling and no floor effects. Modification index was highest in two pairs of error terms: (1) Item 3 “Fresh” and Item 4 “Energetic”, (2) Item 3 “Fresh” and Item 5 “Able to think of new ideas”. Incorporating the corresponding error covariances resulted in satisfactory fit. In view of the error covariances, a seven-item scale with Q3 removed was tested. Table 2 summarizes the fit indices of attempted CFA models. Figure 2 show the standardized coefficients of the seven-item one-factor CFA model.

**Table 2:** Model fit indices in confirmatory factor analysis of the Chinese version of THAT

Model	χ^**2**^ statistic	Degrees of freedom	RMSEA	SRMR	CFI
10-item one-factor	76.13	35	0.11	0.11	0.97
8-item one-factor	40.86	20	0.11	0.10	0.98
8-item one-factor (modified^a^)	22.61	18	0.05	0.07	1.00
7-item one-factor	18.54	14	0.06	0.08	1.00

*RMSEA* root mean square error of approximation, *SRMR* standardized root mean square residual, *CFI* comparative fit index

^a^Incorporated error covariances: Q3 ~ Q4 and Q3 ~ Q5

### Scale reliability and convergent validity

The coefficient omega of the seven-item scale was 0.80 (95% CI: 0.74–0.86). The corrected item-scale correlations ranged from 0.30 to 0.68.

Table 3 assesses the hypothesized association between THAT and the CES-D, PSQI, and AIS. The correlation was generally moderate in a range of 0.40 to 0.45. All correlation coefficients were statistically significant.

**Table 3:** Correlations between Chinese version of THAT score and other subjective scales

	CES-D	PSQI	AIS
7-item one-factor	−0.45^**^	− 0.40^**^	− 0.45^**^

^**^P < 0.01. *THAT* Toronto Hospital Alertness Test, *CES-D* Center for Epidemiological Studies Depression Scale, *PSQI* Pittsburgh Sleep Quality Index, *AIS* Athens Insomnia Scale

## Discussion

This study rigorously translated the THAT into traditional Chinese and evaluated its psychometric performance with a Cantonese speaking population. The unidimensional Chinese version of THAT demonstrated satisfactory reliability and validity in Chinese people, in a Hong Kong setting.

The item non-responses were at most 3%, demonstrating that the Chinese version of THAT was acceptable to the participants. However, the last two items, items 9 and 10, had low factor loadings that reflect their low association with the other items. This is consistent with a previous Canadian study [16]. This may be due to the questionnaire format, as the last two items are negatively worded, whereas the others are positively worded [16]. Individuals usually tend to agree more with positively worded items than negatively worded items [38]. Alternatively, Item 9 “Extra effort was needed to maintain alertness” may not be culturally consistent with a common practice of Chinese individuals, who may choose to rest rather than make an extra effort to maintain alertness [39, 40]. For Item 10 “In a boring situation, I would find my mind wandering”, studies had demonstrated that mind-wandering was not only an indicator of lacking alertness, but it can indicate future-oriented and creative thinking [41]. Furthermore, distinguishing different types or forms of mind-wandering may help explain these new findings [41].

Item 3 contributed to two error covariances that were required for satisfactory fit of the eight-item one-factor model. Firstly, the error covariance between Item 3 “Fresh” and Item 4 “Energetic” may be attributable to similar word meaning. In Chinese culture, a feeling of fresh or energetic is a common description of a good state of spirit and body. Secondly, the error covariance between Item 3 “Fresh” and Item 5 “Able to think of new ideas” may be due to the benefits from a good state of mind, such as positive affect and positive thinking [42]. In general, a state of “Fresh” partly comes from good sleep quality, which plays a critical role in regulating thinking ability, such as divergent thinking ability [43]. Therefore, the higher correlation due to similar meanings of Item 3 with Items 4 and 5 may make it unnecessary. After removing Item 3, the 7-item version fitted well. However, more research is valuable for evaluating Item 3.

The THAT in the Chinese language confirmed previously observed associations of alertness with CES-D, PSQI, and AIS scores, with correlation ranging from 0.40 to 0.45. Depression has been shown to play an important role in regulating alertness [44]. Depressive individuals have lower levels of attention and vigilance, which can be attributed to decreased prefrontal cortex and anterior cingulate cortex (ACC) volume. These areas are partly responsible for processing visceral, effective, and attentional information [45]. Moreover, poor sleep quality also has a relationship with diminished alertness [19]. Poor sleep quality characterized by sleep fragmentation is correlated with sleepiness, and the effective treatment of fragmentation can help reduce sleepiness [46]. In terms of subjective sleep quality, restoration from sleep is well recognized [47], but nonrestorative sleep usually presents with higher sleepiness [48]. Lastly, insomnia is also responsible for impaired alertness [9]. Insomnia patients usually have poor sleep quality which can explain the relationship with alertness [49].

Despite our rigorous translation and psychometric assessment of the Chinese version of THAT, several limitations need to be mentioned. First, our sample was relatively small and larger sample size would be good to further explore the scale structure of the THAT and assess its measurement invariance across groups, such as sex. Second, it would also be desirable to examine test-retest reliability. Lastly, the AIS has not been standardized validated in the Hong Kong Chinese adults. Nevertheless, the seven-item THAT in the Chinese language was found to be reliable and valid for assessing individual alertness in the present study.

## Conclusions

The Chinese version of THAT is a reliable and valid instrument which can help future research in distinguishing and treating alertness without time-spending or resource-spending measurements.

## Data Availability

No additional data are available.

## Abbreviations

AIS: Athens Insomnia Scale
CES-D: Center for Epidemiological Studies Depression Scale
CFA: Confirmatory factor analysis
CFI: Comparative fit index
DWLS: Diagonal weighted least squares
MLR: To robust maximum likelihood
PSQI: Pittsburgh Sleep Quality Index
RMSEA: Root mean square error of approximation
SD: Standard deviation
SRMR: Standardized root mean square residual
THAT: Toronto Hospital Alertness Test
ZOGIM-A: ZOGIM Alertness
